# The Persian adaptation of Baddeley’s 3-min grammatical reasoning test

**DOI:** 10.1186/s41155-017-0070-z

**Published:** 2017-08-08

**Authors:** Purya Baghaei, Fahimeh Khoshdel-Niyat, Mona Tabatabaee-Yazdi

**Affiliations:** 1 English Department, Mashhad Branch, Islamic Azad University, Mashhad, Iran; 2 Purya Baghaei, Islamic Azad University, Ostad Yusofi St, Mashhad, 91871 Iran

**Keywords:** Grammatical reasoning test, Fluid intelligence, Validity, Test adaptation

## Abstract

Baddeley’s grammatical reasoning test is a quick and efficient measure of fluid reasoning which is commonly used in research on cognitive abilities and the impact of stresses and environmental factors on cognitive performance. The test, however, is verbal and can only be used with native speakers of English. In this study, we adapted the test for application in the Persian language using a different pair of verbs and geometrical shapes instead of English letters. The adapted test had high internal consistency and retest reliability estimates. It also had an excellent fit to a one-factor confirmatory factor model and correlated acceptably with other measures of fluid intelligence and participants’ grade point average (GPA).

## Background

The 3-min grammatical reasoning test (Baddeley, 1968) is a widely used measure of fluid intelligence (*Gf*). It is administered in groups, does not need any training or equipment for administration, and requires only 3 min to conduct. It has a straightforward structure and can be scored easily, making it optimal for research on cognitive abilities and the impact of environmental factors, stress, or drugs on human performance.

The 3-min grammatical reasoning test (Baddeley, 1968) was originally developed to measure the effects of “nitrogen narcosis,” the drowsy state experienced by deep-water divers as a result of breathing under high-pressure depths, on divers’ mental capability (Baddeley, 1999). Since the test had to be completed under water in extremely limited time, it had to be very short. Baddeley came up with a grammatical reasoning test using 64 statements that described the order of presentation of two letters “A” and “B” with the verbs “precede” and “follow” using two forms of negative and positive and two voices of passive and active. The idea was based on psycholinguistic research at the time which demonstrated that active and positive sentences are processed more quickly than negative and passive sentences (Roberts, 1968; Wason, 1961). The five binary conditions of proceed/follow, positive/negative, active/passive, true/false, and A or B mentioned first resulted in 32 possible permutations which served as items. Sample items were like “A follows B (AB),” “B precedes A (BA),” “A does not follow B (AB),” and “B is not proceeded by A (AB)” where respondents had to mark whether each statement is a true or false description of the order of the letters presented. The test proved to be very sensitive even at the shallowest depth where nitrogen narcosis was believed to exist and robust against practice. Retest reliability was reported to be 0.80, and evidence of validity was provided by reporting a correlation coefficient of 0.59 with the British Army verbal intelligence test (Baddeley, 1968).

Over the years, Baddeley’s 3-min grammatical reasoning test has come to be known as a quick measure of fluid intelligence (Baudson & Preckel, 2016). Since administering long tests is not practical in research when other measures are also administered, the grammatical reasoning test serves as a very practical and time-efficient measure of *Gf*. For instance, Baddeley’s grammatical reasoning test (along with Raven’s matrices) has been included in the NeuroCognitive Performance Test (Lumos Labs, Inc.), a short, web-based cognitive assessment tool to assess functioning in working memory, fluid and logical reasoning, and some other cognitive abilities (Morrison, Simone, Ng, & Hardy, 2015).

Evidence for the validity of the test as a measure of intelligence has been accumulated by reporting high correlations with other tests of intelligence. For instance, Hartley and Holt (1971) reported a correlation of .70 between the grammatical reasoning test and the AH4, a group test of general intelligence in children. Chamorro-Premuzic and Furnham (2008) employed the grammatical reasoning test as a measure of *Gf* along with the Wonderlic Personnel Test (Wonderlic, 1992) in a study to investigate the roles of personality and intelligence in predicting academic success. Although validating the grammatical reasoning test was not the aim of their research, they reported a correlation of 0.44 between their two measures of intelligence. In another study, Furnham and Chamorro-Premuzic (2006) demonstrated that the grammatical reasoning test correlates with the Wonderlic Personnel Test, Raven’s Advanced Progressive Matrices (Raven, Court, & Raven, 1977), and General Knowledge Test (Irwing, Cammock, & Lynn, 2001) at .65, .39, and .35, respectively. The correlations reported between the grammatical reasoning test and other intelligence tests are almost as high as the correlations between pure nonverbal measures of intelligence.

Being verbal, Baddeley’s 3-min grammatical reasoning test is appropriate for native speakers of English and has mainly been used in English populations (Silver, Phelps, & Dunlap, 1989). Recently, however, other researchers have made an attempt to adapt and translate the test in other languages. Baudson and Preckel (2016) have adapted the test in German. Since the verbs “precede” and “follow” do not work in German in the passive voice, they used two other verbs, namely, “reject” and “prefer” along with the shapes of a circle, a triangle, and a square. The items explain the distances among the shapes. The triangle is always in the middle, and the circle and the square are located in different distances to the triangle. If the circle is close to the triangle and the square is further away from the triangle, the “triangle prefers the circle” or “the square is rejected by the triangle”. Baudson and Preckel (2016) came up with 64 affirmative, negative, passive, and active sentences describing the proximity of the circle and square to the triangle. They reported that the test has structural validity, is reliable, and correlates acceptably with other measures of fluid intelligence.

Another attempt to translate the grammatical reasoning test was made by Karwowski et al. (2016) who translated the test into Polish. In a study on the relationship between creativity and intelligence, they employed the Polish translation of the grammatical reasoning test as a measure of intelligence among numerous other measures of intelligence. Karwowski et al. do not provide any details about the translation and validation process of the test nor do they report any correlation between the grammatical reasoning test and their other measures of intelligence. They only report Cronbach’s alpha reliabilities of .93 and .73 for the test in two different samples.

Due to the lack of a brief and quick measure of fluid reasoning in the Persian language for applications in research and clinical trials, we decided to adapt Baddeley’s grammatical reasoning test in Persian. The psychometric characteristics of the grammatical reasoning test have been studied using classical methods, i.e., by demonstrating its retest consistency and its correlation with other measures of *Gf*. So far, no study has addressed the validity of this widely used measure using modern latent trait models. In this study, we adapt the grammatical reasoning test in the Persian language and investigate the structure of the Persian adaptation by examining its fit to a confirmatory factor model. We also examined its association with other measures of fluid reasoning and academic achievement.

## Methods

### Participants

The participants in this study were 196 (79 female, 107 male, *M*_age_ = 22.71, SD = 7.99) undergraduate Iranian students in different fields of study in several Iranian universities. The native language of all the participants was Persian. Eight intact classes with sizes 19 to 31 were tested in their regular class times. All the participants took the Persian adaptation of Baddeley’s grammatical reasoning test (PAGRT). However, due to the limitations in time and resources, testing all the participants on all the criterion measures was not possible. Therefore, we administered each criterion measure to only two of the classes. Selection of the classes for each criterion measure was done randomly. Participation was voluntary, and students were given course credit for their cooperation. The study was approved by the ethics board of the university.

### Measures

#### The Persian adaptation of the 3-min grammatical reasoning test

A direct translation of the original Baddeley’s (1968) grammatical reasoning test into Persian was not possible as the verbs “precede” and “follow” do not work in Persian in the passive voice. For this reason, two other verbs “inscribe” and “circumscribe” along with the shapes of a square inside a circle and a circle inside a square were used. Some sample items are presented in Table 1.

**Table 1:** Sample items of the adapted grammatical reasoning test

Item		True	False
The square inscribes the circle.			
The circle is inscribed by the square.			
The square does not circumscribe the circle.			
The circle is not circumscribed by the square.			

The five binary conditions of inscribe/circumscribe, passive/active, negative/positive, true/false, and square mentioned first or circle mentioned first resulted in 32 combinations which comprised the 64 items of the test. As in Baddeley’s (1968) original test, each statement was used twice in the test. The statements were interspersed randomly all across the test paper. For the Persian adapted grammatical reasoning test, the time limit was set at 3 min for the entire test.

### Raven’s Advanced Progressive Matrices

A subsample of the participants (*n* = 52) took the short form of Raven’s Advanced Progressive Matrices. The short form of Raven’s test contains 12 items of the original 36-item test. The 12 items of the short form were selected by Arthur and Day (1994) on the basis of rigorous psychometric criteria with the aim of reducing administration time. The test is a measure of fluid intelligence. The Cronbach’s alpha reliability of the 12-item measure in this study was .71.

### Cattell’s Culture Fair Tests

Another subsample of the participants (*n* = 54) took Cattell’s Culture Fair Tests (CCFT, Cattell, 1973) which is a measure of *Gf*. The four paper and pencil subtests of scale 3, i.e., *series*, *classifications*, *matrices*, and *conditions*, were employed. According to the standard instructions for administering the test, 2.50 to 4 min were allotted for each subtest (time varies for each subtest). When the allotted time for a subtest ran out, participants had to stop and move on to the next subtest. The sum of all the correct items on the four subtests constituted the CCFT score. The Cronbach’s alpha reliability of the test was .75.

### Verbal analogies

A small portion of the participants (*n* = 46) took a verbal analogies test. The verbal analogies test contained 41 four-option multiple choice items. The test was constructed and validated by Tabatabaee-Yazdi (2017) to be used in cognitive research in the field of second language acquisition. The validity of the test was confirmed by demonstrating its fit to the Rasch latent trait model (Rasch, 1960/1980). The test had a correlation of .53 with the short form of Raven’s Advanced Progressive Matrices (Tabatabaee-Yazdi, 2017). Kuncel, Hezlett, and Ones (2004) state that verbal analogies are a measure of *g*, the general intelligence factor. The Cronbach’s alpha reliability of the test was .77. A sample item follows:

Mason to Wall is as…

1. Artist to Easel
2. Fisherman to Trout
3. Author to Book
4. Sculptor to Mallet

## Results

### Descriptive statistics

Since the adapted grammatical reasoning test was timed, test takers reached only some of its items, obtaining a mean score of 22.89 out of 64 and a standard deviation of 7.46 (range = 7–41). To analyze the test, four subtests were constructed by categorizing the 64 dichotomous items as “Affirmative Active,” “Affirmative Passive,” “Negative Active,” and “Negative Passive.” There were 16 dichotomous items under each classification, and total scores on each of the four subtests were calculated by aggregating the correct replies in each category. The Cronbach’s alpha reliability of the test considering each subtest as a super item (Eckes & Baghaei, 2015) was .91 with a 2-week retest reliability of .76. Table 2 shows the descriptive statistics for the subtests, and Table 3 shows the correlations between them.

**Table 2:** Descriptive statistics for the subtests of PAGRT

Subtest	Mean	SD	Skewness	Kurtosis	Range
Affir. Act.	6.07	2.06	.06	− .98	2–10
Affir. Pass.	5.00	2.20	.39	− .62	1–11
Neg. Act.	5.32	1.93	.47	− .13	1–11
Neg. Pass.	5.78	2.39	− .13	− .68	1–11

**Table 3:** Correlations between the subtests of PAGRT and their CFA factor loadings

	1	2	3	4
Affir. Act.	.88	.77	.73	.74
Affir. Pass.		.89	.76	.71
Neg. Act.			.84	.67
Neg. Pass.				.81

Note: All correlations are significant at *p* < .001 (two-tailed). CFA factor loadings are in diagonal

### Confirmatory factor analysis

The adapted grammatical reasoning test was validated by fitting the confirmatory factor analysis (CFA) model. Fit of data to a latent trait model is evidence that a latent dimension underlies the test which accounts for the covariation among items (Baghaei & Tabatabaee, 2016; Borsboom, 2008). Amos 23 (Arbuckle, 2014) was used to perform the analyses. Each subtest (Affirmative Active, Affirmative Passive, Negative Active, and Negative Passive) was considered an indicator and a one-factor model with four observed variables was fitted. CFA had an excellent fit to the data, *χ*^2^/*df* = 1.63, CFI = .99, GFI = .99, TLI = .99, and RMSE = .05. The GFI, TLI, and CFI were greater than their cutoff criteria of .90 which indicates that the model adequately accounted for the variance and covariance in the scores. The *χ*^2^/*df* of 1.63 and RMSE of .05 were less than the recommended cutoff values of 3 and .08, respectively. This is evidence of an adequate model specification and a good fit between the model-specified variance/covariance matrix and the population variance/covariance matrix. All parameter values in the model were statistically nonzero (*p* < .001), thus supporting adequate model specification. The examination of modification indices showed no evidence of error correlations. The fit of a one-factor model is evidence that a single ability or trait underlies the test and causes the item responses (Borsboom, Mellenbergh, & van Heerden, 2003).

### External validation

In order to provide external validity evidence for the adapted test, participants’ scores on four independent criteria, namely, Raven’s Advanced Progressive Matrices (short form), Cattell’s Culture Fair Test Scale 3, and a verbal analogies (VA) test, were correlated with performance on the PAGRT. The grade point average (GPA) of a small group of the participants (*n* = 50) was also obtained from the university administration. Table 4 depicts the coefficients of correlation between the PAGRT and the external criteria.

**Table 4:** Correlations between the PAGRT and external criteria

	Raven	Culture Fair Test	Verbal analogies	GPA
PAGRT	*r* = .35 *n* = 52	*r* = .52 *n* = 54	*r* = .50 *n* = 46	*r* = .41 *n* = 50

Note: All correlations are significant at *p* < .01 (two-tailed)

The correlations between the PAGRT and other measures of intelligence are in line with what is reported in the literature. In a study on the relationship between working memory capacity and reasoning ability, for instance, Kyllonen and Christal (1990) correlated a number of working memory tests with reasoning tests and knowledge tests. Among the reasoning tests they used were Baddeley’s grammatical reasoning test and verbal analogies test. They also used a paragraph comprehension test as one of the measures of general knowledge. The grammatical reasoning test correlated at .47 with the verbal analogies test and .20 with paragraph comprehension. It also correlated with Arithmetic Reasoning and Mathematics Knowledge sections of the Armed Services Vocational Aptitude Battery (US Department of Defense, 1984) at .38 and .42, respectively.

## Discussion

Baddeley’s (1968) 3-min grammatical reasoning test is a quick and economic measure of *Gf* which has extensively been used in research on cognitive abilities. The test is an excellent choice in contexts where full-scale IQ measures are difficult to use. However, the verbal nature of the test makes it only appropriate for use with native speakers of English. In this study, we adapted the test to be used in the Persian language.

A direct translation of Baddeley’s 3-min grammatical reasoning test into Persian was not possible as the passive forms of the verbs “follow” and “proceed” cannot be translated into Persian. Therefore, in our adaptation, we used two other verbs, i.e., “inscribe” and “circumscribe” along with the shapes of a square inside a circle and a circle inside a square; respondents had to decide whether the statements (items) were true about the positions of the shapes.

The adapted test had satisfactory internal consistency and retest reliability estimates, and fitted well to a one-factor CFA model which is theoretically viable. Thus, the adapted test measures a unidimensional construct, and respondents’ abilities can be reported by assigning a single score.

The adapted test also showed acceptable evidence of criterion-related validity in several subgroups of undergraduate university students. External validity evidence was provided by demonstrating the association between the Persian adapted grammatical reasoning test and other criteria. The observed patterns of correlations were in line with what other researchers have reported in the literature. The PAGRT showed acceptable coefficients of correlation with Raven’s APM, Cattell’s Culture Fair Tests, and verbal analogies test. The lower correlation with Raven’s APM was probably because we used the short form of the APM which contains only 12 items. Low reliability (alpha = .71) and restriction of range on Raven’s test could be a reason for the lower correlation between this criterion and PAGRT.

The PAGRT also moderately correlated with students’ GPA which is consistent with the existing literature. Research has demonstrated that there is a low to moderate correlation between *Gf* and GPA. For instance, Laidra, Pullmann, and Allik (2007) showed that Raven’s Standard Progressive Matrices correlated with GPA between .32 and .54 for school children in grades 2 to 12 (age range from 7 to 19). As grade increased, the magnitude of correlations diminished. Similarly, Di Fabio and Palazzeschi (2009), investigating the predictors of scholastic success among Italian high school students, found a correlation of .32 between Raven’s Advanced Progressive Matrices and GPA. Other researchers have found a low to medium correlation between *Gf* and GPA (Di Fabio & Busoni, 2007; Steinmayr, Ziegler, & Träuble, 2010).

Findings showed that the PAGRT correlated higher with the CCFT than with Raven’s test. Cattell (1980) expressed his doubts about the Raven matrices as a good measure of *Gf*. He argued that a good measure should employ several different subtests “to wash out any undue contamination by one specific one” (p. 337). Along the same lines, Jensen (1980) wrote that Raven’s matrices contain specific variance because of using only matrices and states that since CCFT has several item formats, it is not affected by test method variance.

The correlation between PAGRT and CCFT was almost as high as the correlations reported in the literature between purely nonverbal measures of *Gf*. For instance, Conway, Cowan, Bunting, Therriault, and Minkoff (2002) reported a correlation of .57 between Raven’s Standard Progressive Matrices and Cattell’s Culture Fair Test which is very close to the correlation obtained in this study between CCFT and PAGRT (*r* = .52).

The relatively high correlation between the PAGRT, a verbal measure based on reasoning with grammar, and Cattell’s Culture Fair Test, a purely nonverbal measure of *Gf*, is in line with Oller’s (1981) thoughts on the link between language and intelligence. Oller suggests that language is not just a phenomenon related to the social aspect of human life, but “it may be the very foundation of intelligence itself” (p. 466). He supports his argument with evidence from genetics, neurology, and psychometric studies of intelligence tests and concludes that “language at its deepest level may well constitute the very essence of intelligence” (Oller, 1981, p. 490). The close affinity between intelligence and logic was also suggested by Piaget (1947, cited in Oller, 1981). Oller (1981) states that for normal use of language, deep logic is required. This logic is in fact grammar with its rules and constraints which is almost indistinguishable from Piaget’s definition of intelligence. Oller (1981) states that logic is heavily language-dependent; hence, language and intelligence are closely linked.

The current study investigated the reliability and validity of the PAGRT on Iranian university undergraduate students. Assessing the validity and reliability of the test on more diverse populations from other Persian-speaking countries is recommended. The test should also be examined with other population of students, with school children and adults of different ages. Further research should compare the power of PAGRT with other measures of fluid reasoning in predicting outcome measures such as academic performance.

## Conclusions

The findings of the study demonstrated that Baddeley’s (1968) 3-min grammatical reasoning test is adaptable in Persian which confirms its cross-cultural validity. The adapted test, using shapes and a different pair of verbs, in Persian fitted a one-factor CFA model which is theoretically justified. The test had high retest and internal consistency reliability estimates and correlated acceptably with external criteria. All these findings suggest that the adapted test is a valid measure of *Gf* in the Persian language.

The idea of using the verbs “inscribe” and “circumscribe” along with geometrical shapes can also be used in other languages where the translation of the verbs “precede” and “follow” do not work. The chances are high that the verbs inscribe/circumscribe have direct equivalents in many languages, and therefore, translating them into those languages should be less problematic than translating proceed/follow. We strongly encouraged translating the test in other languages and investigating its concurrent validity against standard measures of *Gf*. Further research could also investigate the psychometric properties of the test by examining its fit to item response theory models. To support the utility of the scale across different subpopulations, invariance analysis is also recommended.
